# Hematopoietic Stem Cell Transplantation and Vasculopathy Associated With STAT3-Dominant-Negative Hyper-IgE Syndrome

**DOI:** 10.3389/fped.2020.00575

**Published:** 2020-09-10

**Authors:** Mark J. Ponsford, James Clark, Joel Mock, Mario Abinun, Emily Carne, Tariq El-Shanawany, Paul E. Williams, Anirban Choudhury, Alexandra F. Freeman, Andrew R. Gennery, Stephen Jolles

**Affiliations:** ^1^Immunodeficiency Centre for Wales, University Hospital for Wales, Cardiff, United Kingdom; ^2^Division of Infection, Inflammation, and Immunity, School of Medicine, Tenovus Institute, Cardiff University, Cardiff, United Kingdom; ^3^Department of Interventional Cardiology, University Hospital for Wales, Cardiff, United Kingdom; ^4^Paediatric Haematopoietic Stem Cell Transplant Unit, Translational and Clinical Research Institute, Great North Children's Hospital, Newcastle University, Newcastle upon Tyne, United Kingdom; ^5^Department of Interventional Cardiology, Morriston Hospital, Swansea Bay University Local Health Board, Swansea, United Kingdom; ^6^Laboratory of Clinical Immunology and Microbiology, National Institute of Allergy and Infectious Diseases, National Institutes of Health, Bethesda, MD, United States

**Keywords:** Job's syndrome, STAT3 transcription factor, ST elevation myocardial infarction, coronary vessels, thrombosis, cardiovascular diseases, hematopoietic stem cell transplantation, counseling

## Abstract

Dominant negative mutations in the transcription-factor *STAT3* underlie the rare primary immunodeficiency Job's syndrome. Allogeneic Hematopoietic Stem Cell Transplantation (HSCT) has shown promise in correction of the underlying immunological defect, with one report suggesting HSCT can prevent development of wider connective tissue complications. Here, we report the case of a 26 year old male who developed an acute ST-elevation myocardial infarction due to coronary artery ectasia and thrombosis, occurring despite pediatric allogeneic HSCT for STAT3-HIES and a predicted 10-year conventional cardiovascular risk of 0.1%. Vasculopathy associated with STAT3-HIES may persist or arise following HSCT and can precipitate life-threatening complications. This has implications for counseling and vascular surveillance, and highlights the need for further studies to determine the risk, pathogenesis, and optimal management of the vasculopathy associated with STAT3-HIES.

## Introduction

“Job's syndrome” was first coined in 1966 by Davis et al. to describe the constellation of childhood-onset dermatitis, recurrent staphylococcal “cold” skin abscesses, and chronic sinopulmonary disease accompanied by extreme elevation of immunoglobulin IgE (1). Dominant negative mutations in the transcription factor *STAT3* underlie ~75% of sporadic or autosomal dominant “hyper IgE syndromes” (HIES) (2–5), and give rise to a wide range of multi-system complications (6). These include vascular abnormalities, scoliosis, facial dysmorphism, osteoporosis, and retained primary teeth, accompanied by elevated malignancy risk (6). Defective wound healing aggravates recurrent pulmonary infections, accelerating respiratory decline, and mortality risk (7). Multi-disciplinary care and treatments, including antimicrobial prophylaxis and immunoglobulin replacement therapy (IgRT), is aimed at slowing this decline. Allogeneic hematopoietic stem cell transplantation (HSCT) has been used in a small but growing number of cases, prompted by hematological malignancy or infection-burden and parenchymal lung damage despite conventional therapy. These demonstrate a role for HSCT in resolving the immunological deficit and modifying pulmonary decline in selected STAT3-HIES patients (8–11). HSCT has also been suggested to halt or delay development of extra-hematological manifestations of STAT3-HIES “*including neurological, musculoskeletal, and vascular abnormalities*” when conducted in adolescence (12). With increasing interest in the curative potential of HSCT, accurate description of the limits and potential of HSCT is timely (13) and carries significant implications for patients, families, clinicians, and healthcare commissioners. Here, we report in detail the acute myocardial infarction in a patient with genetically-confirmed *STAT3*-HIES, due to thrombosis of an ectatic coronary artery, despite uncomplicated allogeneic HSCT conducted in childhood. We reviewed medical case notes to determine the patient's pre-morbid cardiovascular disease (CVD) risk score, and surveyed the literature to address issues raised by this case concerning HSCT, vasculopathy, and cardiovascular outcomes in relation to STAT3-HIES. The following Medline search terms were used (“myocardial infarction” OR “vasculopathy” OR aneurysm OR rupture) AND (STAT3 OR “signal transducer and activator of transcription 3” OR “Hyper IgE Syndrome” OR “HIES”) on 06-MAY-2020 to identify the state of published knowledge.

### Case Report

A young Caucasian male was diagnosed with Job's syndrome age 6 years on the background of neonatal onset dermatitis with boils, recurrent sino-pulmonary infections, and IgE >6,000 kU/L (normal <56). *STAT3*-HIES was confirmed on the basis of an NIH score of 76 (14) and Sanger sequencing demonstrating heterozygous state for 1144C>T (R382W); a common mutation interfering with the DNA-binding domain of *STAT3*. Despite antimicrobial prophylaxis and IgRT, he experienced frequent severe broncho-pneumonias with progressive bilateral bronchiectasis. On this basis, he underwent myeloablative HSCT aged 13 years with Alemtuzumab, Fludarabine, Melphalan conditioning. Subsequent to HSCT, he showed 100% donor chimerism in all hematopoietic cell lineages, normalization of vaccination responses and marked decline in serum IgE from 8,778 kU/L to 176.0 kU/L (8). IL17A production and Th17 subsets normalized post HSCT (8). Following an initial diagnosis of delayed puberty secondary to chronic childhood illness, he was commenced on testosterone replacement therapy under specialist endocrine monitoring. He was able to cease immunoglobulin replacement and graft vs. host disease prophylaxis, and gained full-time employment, maintained on prophylactic azithromycin, fluticasone nasal spray, vitamin D, with annual zoledronate therapy. He remains under care of the All Wales HSCT multi-disciplinary team.

**Figure 1:**
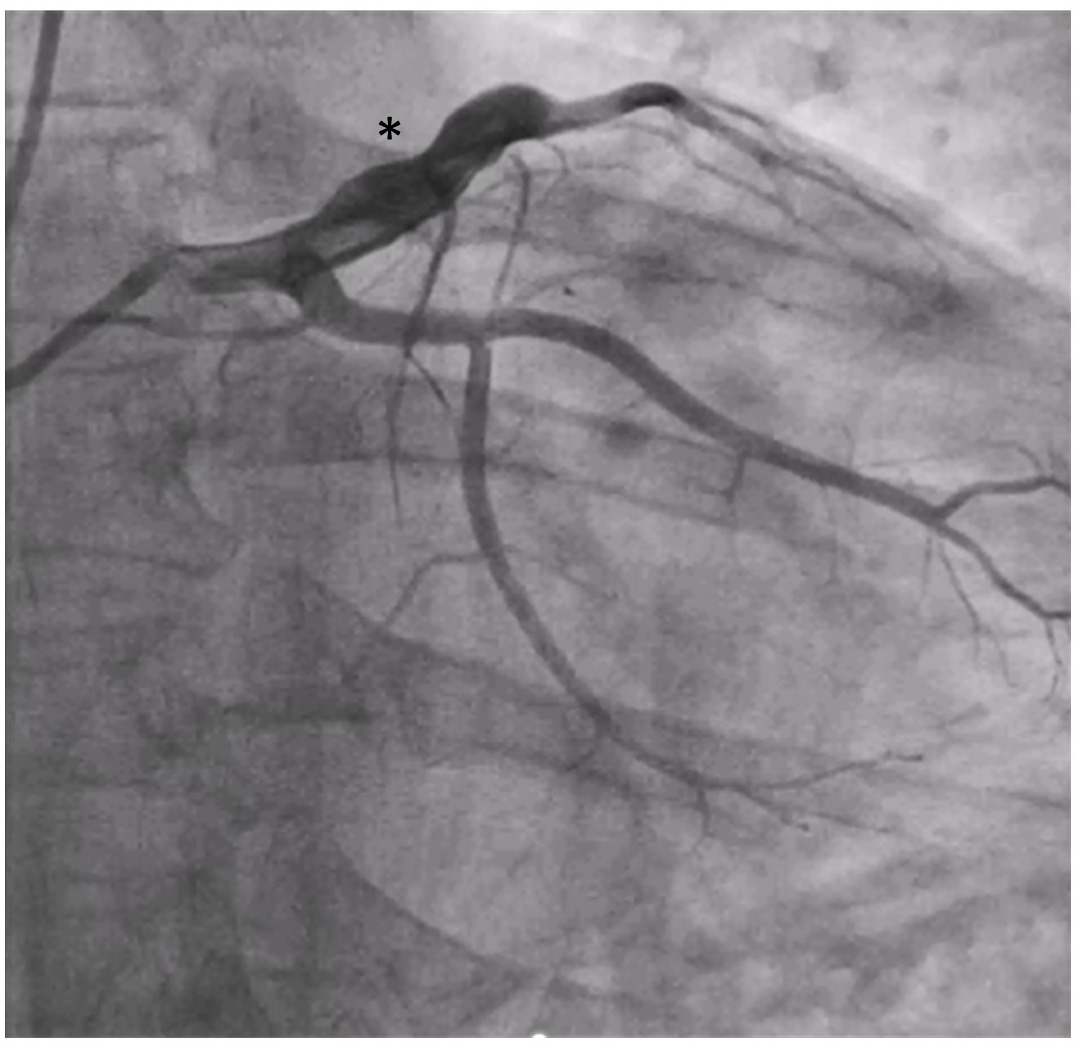
Proximal ectasia (*) revealed by angiography.

**Figure 2:**
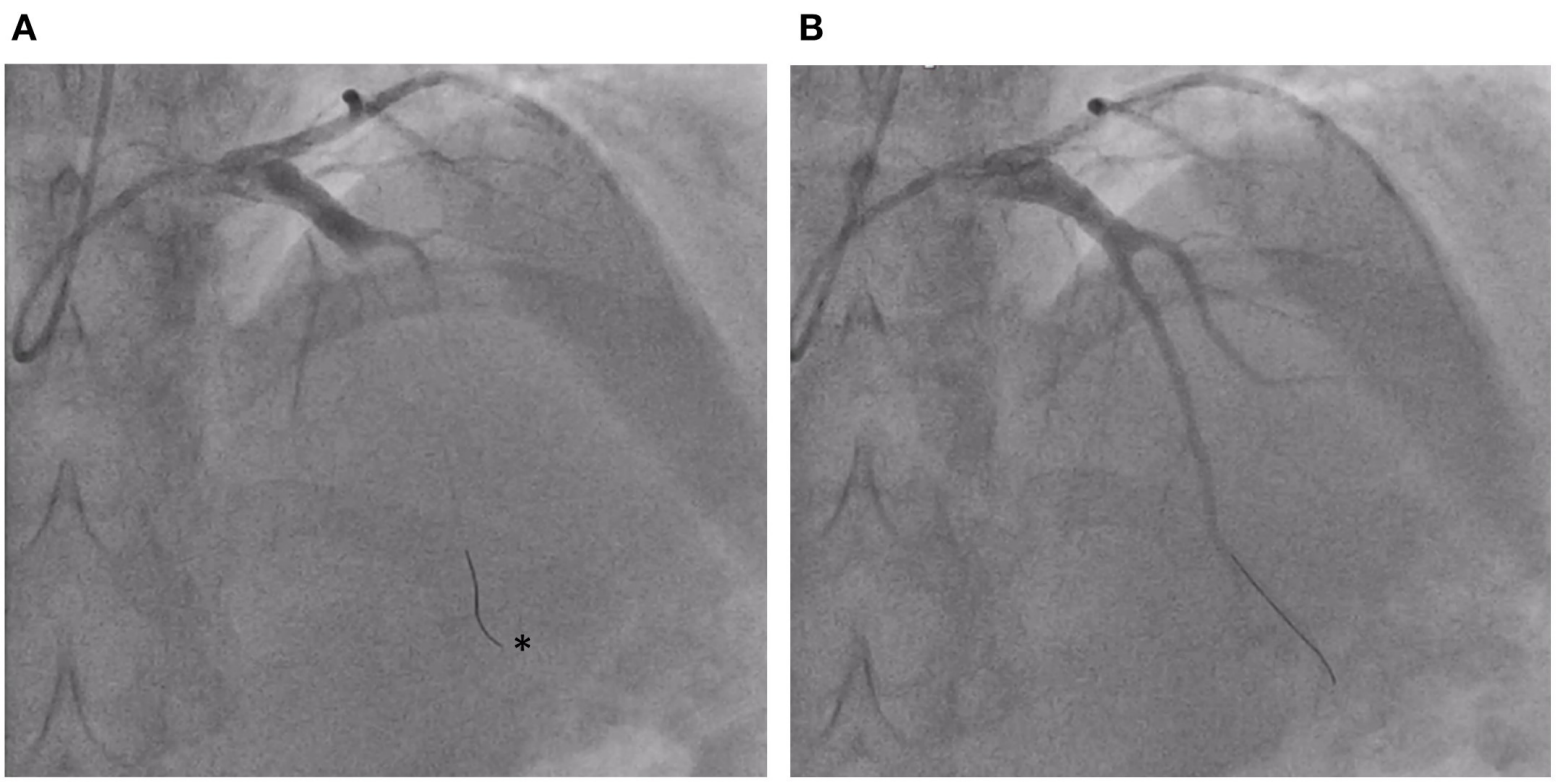
**(A)** Catherization of the left coronary circulation reveals occlusion of left anterior descending (LAD) coronary artery. The radio-opaque tip of the angioplasty wire (*) can been seen in distal LAD having traversed the site of thrombotic occlusion in mid LAD. **(B)** Flow restoration following intra-coronary thrombolysis and mechanical thrombus aspiration.

Over a decade following HSCT, he developed sudden-onset central chest pain waking him from sleep. Emergency department electrocardiogram showed widespread anterior ST-elevation, associated with a 3 h troponin rise from 26 to 43,249 ng/L. Full blood count, CRP, and ESR were within normal limits with a total cholesterol of 4.8 mmol/L (<5), non-fasted LDL-cholesterol 3.3 mmol (<3.0), HDL-cholesterol 1.1 mmol/L (>1.0), and triglyceride 0.8 mmol/L (<2.0). At routine clinic review 1 month prior, he was normotensive (126/84 mmHg) with BMI 23.1 kg/m2. There was no specific family history of premature CAD, arrhythmia, or sudden cardiac death and no prior history of Kawasaki's disease. Aged 26 years, as a life-long non-smoker with no history of illicit drug use, his predicted 10-year risk of heart attack or stroke based on UK-population datasets (QRISK3) was 0.1% (15). He received a diagnosis of ST-elevation myocardial infarction (STEMI) and underwent urgent primary percutaneous coronary intervention within 12 h of symptom onset. Angiography revealed proximal ectasia leading on to mid-vessel occlusion of his left anterior descending (LAD) coronary artery, with otherwise unobstructed coronary vessels (Figure 1; Supplementary Video 1). Intra-coronary thrombolysis using tissue plasminogen activator along with mechanical aspiration of thrombus led to restoration of flow in the LAD (Figure 2; Supplementary Videos 2, 3). No underlying stenosis at the point of the initial vessel occlusion was noted, hence thrombus formation in the ectatic proximal LAD with distal embolization and occlusion of the mid LAD was felt to be the likely mechanism of his presentation with STEMI. Dual pathway blockade (coagulation and platelet inhibition) was initiated to offer protection against recurrence of coronary thrombotic occlusion (16, 17) with aspirin 75 mg for 3 months, rivaroxaban 2.5 mg twice daily for 3 years and clopidogrel 75 mg long term. Atorvastatin, candesartan, bisoprolol, ivabradine, and spironolactone were also initiated. Echocardiography 1 week post-STEMI showed moderate reduction in left ventricular systolic function with anteroseptal and apical akinesis (Supplementary Video 4). At 12 months, Simpson's biplane ejection fraction remains reduced at 41%, with aneurysmal dilatation of the ventricular apex and persistent regional wall motion abnormalities consistent with mild-moderate systolic impairment. Contrast computed tomography (CT) revealed a bovine aortic arch (a common variant), but no evidence of intra-cranial arterial aneurysms. Examination of the retinal vasculature was also normal.

### What Is the Significance of Vasculopathy in STAT3-HIES?

Concerns around vasculopathy in AD-HIES first emerged in 2007, after report of subarachnoid hemorrhage and myocardial infarctions in relatively stable patients (18). A systematic review conducted in 2010 identified vascular abnormalities in a total of 8 patients with clinically-defined AD-HIES patients from the international literature. Abnormalities included aortic aneurysm (*n* = 1), coronary aneurysms (*n* = 3), congenital patent ductus venosus (*n* = 2), and intracranial vascular abnormalities (*n* = 2). Mortality in 3 of these patients followed aneurysm rupture, myocardial infarction, or thrombotic stroke, with additional stroke-related morbidity (7, 19). Five patients with a clinical diagnosis of AD HIES are described in a Taiwanese cohort. One death occurred due to acute myocardial infarction with dilated coronary artery in a 13 year old male (20). Additional case reports describe symptomatic aortic and cerebral aneurysms in pediatric (21, 22) and adults but with limited genetic and follow-up information. Subsequent cross-sectional studies within genetically-defined STAT3-HIES US (National Institute for Health) and French cohorts have provided convincing evidence for widespread “vasculopathy” in STAT3-HIES. In 59 patients imaged from infancy to adulthood (8 to 57 years), intracranial aneurysms were present in 20%; coronary artery abnormalities (tortuosity, ectasia, or aneurysm) in 70%; and brain abnormalities (white matter hyperintensities, lacunar lesions, and atrophy) in 95% (23–25). Rates of coronary artery tortuosity increased with age (23). Deep venous thrombosis events were also reported in 3/21 patients (aged 28–39), with 2 of these also experiencing pulmonary embolism (25).

The clinical significance and optimal approach to such frequent and widespread vascular abnormalities remains an area of active investigation. Cerebral white matter hyperintensities are associated with an increased risk of dementia in the general population (26). Detailed neurocognitive behavioral profiling in STAT3-HIES has revealed subtle relative impairment of memory and executive functioning measures, despite average to high average overall cognitive function (24). Antiplatelet therapy has been suggested to reduce the risk of coronary artery thrombosis and cerebral microvascular disease (25), however may actually be harmful given prior reports of pulmonary hemorrhage-associated mortality (7). Similarly, an international consensus on approach to intra-cranial aneurysms remains elusive. The detection rate of 20% in STAT3-HIES matches that found by the Familial Intracranial Aneurysm (FIA) study (27). It is relevant to consider this benchmark, given the FIA study only offered vascular screening to those perceived as “high risk” for aneurysm detection (i.e., first-degree relatives of those affected by intra-cranial aneurysm, aged ≥30 years of age, with smoking and/or hypertensive history). Of these, 10% subsequently underwent surgical or endovascular intervention (27). Vascular imaging studies in 59 patients within STAT3-HIES led to a similar proportion of asymptomatic aneurysms being put forward for endovascular coiling (23, 25). How patients, families, and clinicians affected by STAT3-HIES currently perceive and approach vascular risk in routine clinical practice is unclear. A recent report from the US Immunodeficiency Disease Network concerning 85 patients indicates a low uptake of vascular screening in asymptomatic STAT3-HIES patients (<10%) across the US and Canada, outside of the NIH. Aneurysm rupture rates and outcomes in this rare disease population will be important–if challenging–to quantify, as this information is vital to inform discussions concerning interventional approaches. Endovascular coiling or neurosurgical clipping both carry a risk of death or disability, up to 5% (25). Peri-procedural risk is further heightened when performed following acute rupture (25), suggesting this decision is best made early. The consequence of delay is illustrated by the rupture of a giant basilar artery aneurysm in one individual with Job's, causing death shortly prior to the scheduled endovascular procedure, aged 35 years. Additional approaches to modify the risk of aneurysms development and rupture are therefore desirable. Detailed *in vivo* sonographic assessment of seemingly normal carotid vessels in STAT3-HIES patients revealed reduced intima-media thickness (IMT) and subtly increased internal carotid diameter, relative to healthy controls. Circumferential wall stress correlated with frequency and severity of vasculopathy, unlike genotype or NIH Score (a surrogate for degree of multi-system involvement) (25). Under laminar blood flow conditions, circumferential wall stress (CWS) increases proportionally to mean arterial blood pressure, highlighting both a potential biomarker and modifiable risk factor. Hypertension in the NIH cohort appeared more common than expected for age, with an overall incidence of 42% despite a mean age of 31 (23), but was not a clear driver within the normotensive French cohort (25).

### What Evidence Is There That HSCT Can Ameliorate Vascular Pathology?

A clearer understanding of the underlying pathophysiology of vasculopathy in STAT3-HIES will help identify new therapies, and address if HSCT might feasibly be expected to halt their development. In the context of a hypertensive (angiotensin II-driven) murine model of abdominal aortic aneurysm, inhibition of IL-17A signaling promotes inflammatory T-cell infiltration and drives aneurysm growth and rupture (25). Deficiency of IL-17 producing Th17 cells is a pathognomonic feature of STAT3-HIES (28), and their restoration suggests a plausible mechanism by which HSCT might ameliorate non-haematopoietic vascular manifestations. Immune reconstitution with normalization of IgE and detectable levels of IL17+ CD4+ T-cells are consistent features across published HSCT case series (8, 11, 12, 29), including by Goussetis et al. This group reported follow-up of 2 unrelated males with STAT3-HIES following myeloablative matched-sibling HSCT aged 15 and 16 years for high grade Non-Hodgkin Lymphoma (12). Both carried pathogenic mutations in the DNA-binding domain of STAT3 (R382Q or R382W). Follow-up until the ages of 29 and 26 years (respectively), failed to detect any coronary artery aneurysms or brain lesions; leading the authors to suggest HSCT “*cures patients with autosomal dominant hyper-IgE syndrome*.”

This contrasts with our patient's experience of life-threatening coronary artery thrombosis despite undergoing allogeneic HSCT at younger age, with similar mutation type and follow-up duration. Several possibilities may explain these contradictory outcomes, notably the limited sample size, disease-phenotype variability, and distinct HSCT indication and conditioning. We suggest differences in vascular imaging methodology are also important to highlight. Echocardiography was used to screen for coronary artery abnormalities in the prior report (*personal communication: Drs Oikonomopoulou & Goussetis, January 2020*), whereas ectasia was recognized in our patient following invasive coronary angiography. These approaches differ in sensitivity, with echocardiography becoming progressively less accurate for coronary artery imaging with age (30). Coronary artery multidetector computed tomography (CT) angiography is increasingly preferred in adults, and is part of the screening for STAT3-HIES patients over 30 years of age under care of the NIH (https://clinicaltrials.gov/ct2/show/NCT00006150). Magnetic resonance (MR) imaging offers an non-ionizing alternative, and enables additional *in vivo* coronary vessel wall (VW) imaging in patients able to tolerate this procedure, and is utilized for screening and follow-up of the majority of the STAT3-HIES patients followed at NIH (31).

### What Is the Primary Driver for Vascular Abnormalities in STAT3-HIES?

Infection susceptibility is a core diagnostic feature of primary immunodeficiency disorders. Wider causes of hyper IgE syndromes, such as recessive DOCK8 and X-linked WASP deficiency [reviewed Ponsford et al. (32)] are distinctive for marked viral susceptibility and high rates of childhood-onset complications including vasculitis, encephalitis, and stroke-related morbidity/mortality (33). In this genetic-setting, allogenic HSCT clearly offers significant benefit (34), suggesting correction of infection susceptibility is relevant. Limited post-mortem studies in genetically-confirmed STAT3-HIES have provided mixed results in relation to infective triggers. Opportunistic fungal infection (*Scedosporium prolificans or Aspergillus fumigatus*) were identified within the brains of 2 of 6 patients postmortem (7). Notably, these patients also had history of cystic lung disease and active pneumonia at time of death. Retrospective analysis in this setting maybe vulnerable to the *cum hoc ergo propter hoc* (*with this, therefore because of this)* fallacy. Detailed neuropathological examination of a single patient who died from giant basilar artery aneurysm rupture, in the absence of overt fungal pneumonia, identified no evidence of vasculitis or inflammation within the vessel wall of aneurysmal and non-dilated cerebral blood vessels (25, 35). Furthermore, infective causes of aneurysms were not apparent in any of the 59 STAT3-HIES patients imaged (23, 25). These observations do not exclude an infective etiology for vasculopathy in STAT3-HIES, but are suggestive of an intrinsic role for STAT3 in vascular health. This is supported by other monogenic disorders presenting with similar connective tissue abnormalities but without marked infection susceptibility. Loeys-Dietz syndrome (LDS) is caused by autosomal dominant mutations in the transforming growth factor β (TGF-β) receptor pathway. Affected individuals present with atopic tendency and a Marfan-like syndrome that includes familial thoracic aortic aneurysms associated with increased TGF-β signaling within the vessel wall (36). The role of TGF-β1 as a master regulator of matrix metalloproteinases (37) also fits with the dysregulation of these mediators of extracellular remodeling observed in STAT3-HIES (38). Further research at this interface of atopic and syndromic disease has revealed a molecular link between STAT3 and TGF-β (39). The ERBB2-interacting protein (ERBIN) allows STAT3 to negatively regulate TGF-β; loss of ERBIN leads to excessive TGF-β signaling and predicts similar in STAT3 deficiency states. Exaggerated TGF-β protein expression has indeed been documented within post-mortem cerebral arteries in STAT3-HIES (25). Hypertension, *via* biomechanical forces such as shear stress, has also been shown to promote oxidative stress and heighten TGF-β expression during pathological arterial remodeling (40). If correct, a tissue intrinsic role for STAT3 has several implications. *Firstly*, it suggests modulation of TGF-β signaling represents a promising target for therapeutic modulation. *Secondly*, it implies that correction of the hematopoietic stem compartment alone (*via* HSCT or current gene therapy approaches) cannot correct dysregulated signaling due to pathogenic *STAT3* carried within the vascular endothelial and smooth muscle cells. This is consistent with the events observed our patient, and leads to the next question relevant to our case.

### What Role Does STAT3 Play in the Heart?

Convergent evidence supports a critical role for STAT3 in cardiac function. Targeted deletion of murine *Stat3* within cardiomyocytes (cardiac-*Stat3*^−/−^) has been used to circumvent embryonic mortality of complete knockout, and facilitate assessment of its cardiac-intrinsic roles. Cardiac-*Stat3*^−/−^ mice spontaneously develop heart dysfunction with advancing age associated with a dramatic increase in cardiac fibrosis (41). Of particular relevance to our patients' clinical presentation, outcomes following ischemic-reperfusion injury or permanent left coronary artery ligation are significantly worsened relative to wild type (WT) littermates. Following complete ligation, progressive decline in left ventricular systolic function in these mice was accompanied by massive fibrosis, dilatation, and symptomatic heart failure. At 6-months, mortality in cardiac-*Stat3*^−/−^ mice was 100%, compared to 32% in the WT group. Heterozygotes expressing reduced levels of protein appeared unaffected (42). Together, this is consistent with a critical cardioprotective role for *STAT3 within the myocardium*, that is sensitive to gene dosage effects. Additional complexity exists with regard to myocardial healing (43) and thrombosis risk, given impaired neutrophil chemotaxis (42) and dampened platelet activation (44) accompany STAT3-HIES. Thus, the significance of dominant negative *STAT3* mutations in the context of aging and ischaemic insult have yet to be systematically determined. Fortuitously, a murine model carrying a common human *STAT3* pathogenic mutation is now available to address this (45). This recapitulates multiple aspects of human pathology, including extreme elevation of IgE, Th17 cell deficiency, and bacterial infection susceptibility. In this setting, immune reconstitution does not fully correct the *in vivo* susceptibility of these animals to bacterial infection (45), nor rescues cutaneous wound healing defects (46). This is echoed by real-life experience of HSCT, which suggests a reduced infection frequency but ongoing abnormal pulmonary remodeling in terms of pneumatocele development (8, 11). It remains to be seen how/if this transgenic model recapitulates wider aspects of human vasculopathy such as tortuosity and dilatation that will allow dissection of the contribution of infective and environmental stimuli and support pre-clinical evaluation.

## Future Directions

Our understanding of the pathogenesis of vasculopathy in Job's is shaped by developing model systems and detailed clinical observation of Nature's own experiments. The study of rare disease frequently necessitates collaboration to achieve this. An international survey *via* the European Society for Immunodeficiencies (ESID) will shortly be commencing to address clinical knowledge gaps identified in this report (Table 1). We hope this case will encourage readers involved with care for patients with STAT3-HIES to engage with this, thus supporting the most representative insight to natural history of STAT3-HIES to date. Epidemiological information provided is intended to help inform clinical decision making, and is likely to prove timely given advances in precision medicine. Analysis of prospective longitudinal vascular imaging pre- and post-HSCT will be required to definitively answer the potential and limitations of this therapy. A clearer understanding of molecular pathology may support targeted therapeutic strategies, promising what current approaches have yet to convincingly deliver: a holistic therapy for the multi-system complications afflicting those with STAT3-HIES. Whilst TGF-β appears a relevant target, given the range of complex signaling pathways integrated *via* STAT3, additional “master regulators” of interest are also likely to emerge. The creation of STAT3-HIES patient-derived pluripotent stem-cell lines (47) now permits unprecedented insights to the downstream effects of dysregulated STAT3 signaling on cellular transcriptional networks, across a range of relevant human cell types. This approach has recently identified deficiency of hypoxia-inducible factor (HIF1α) as central to defects in extracellular matrix remodeling and angiogenesis (48). Furthermore, use of the HIF1α stabilizing medications Dimethyl Fumarate and Daprodustat normalized wound healing in the STAT3-HIES mouse model *in vivo*, and promoted differentiation of naïve CD4+ T-cells isolated from STAT3-HIES patients into Th17 cells *ex vivo* (48). Careful evaluation will be required to assess if this approach can be safely translated into the clinic. Targeted therapies have already shown tremendous benefit in other monogenic disorders in the field of primary immunodeficiency (49, 50), and promise an exciting new chapter in the book of Job.

**Table 1:** Key learning points and clinical knowledge gaps highlighted by this case.

**Learning points**	**Clinical knowledge gaps**
• Vascular pathology associated with STAT3-HIES can cause life threatening illness. • Hematopoietic stem cell transplantation (HSCT) does not appear to prevent this complication.	• What is the natural history and clinical significance of vasculopathy? • Should vascular screening be offered to patients with STAT3-HIES? • Does impairment to cutaneous and lung healing extend to other (cardiac and cerebral) tissues? • What are the long-term sequelae of cerebral white matter hyperintensities in the aging STAT3-HIES patient group? • Can a molecularly-targeted approach normalize STAT3 signaling pathways across tissues?

## Data Availability Statement

All datasets generated for this study are included in the article/Supplementary Material.

## Ethics Statement

Ethical review and approval was not required for the study on human participants in accordance with the local legislation and institutional requirements. The patients/participants provided their written informed consent to participate in this study. Written informed consent was obtained from the individual(s) for the publication of any potentially identifiable images or data included in this article.

## Author Contributions

MP conceived the article, conducted the literature review, and wrote the first draft of the manuscript. AF, AG, and SJ contributed to conception and design. JC, JM, AC, MA, TE-S, and PW contributed additional clinical data. All authors provided critical revision and approved the final version.

## Conflict of Interest

SJ has received support from CSL Behring, Shire, LFB, Biotest, Binding Site, Sanofi, GSK, UCB Pharma, Grifols, BPL SOBI, Weatherden, Zarodex, and Octapharma for projects, advisory boards, meetings, studies, speaker, and clinical trials. TE-S has received educational support, project support, advisory board fees, speaker fees, and/or clinical trial support from Biotest, CSL, LFB, Mylan, Novartis, Shire, and Werfen. The remaining authors declare that the research was conducted in the absence of any commercial or financial relationships that could be construed as a potential conflict of interest.

## Supplementary Material

Supplementary Video 1 Proximal Coronary Ectasia on coronary angiography.

Supplementary Video 2 Occlusion of Left Anterior Descending (LAD).

Supplementary Video 3 Post clot aspiration and thrombolysis.

Supplementary Video 4 Echocardiogram 1-week post-infarction.
