# Supplementary material for: Hematopoietic Stem Cell Transplantation and Vasculopathy Associated With STAT3-Dominant-Negative Hyper-IgE Syndrome
Source: Front Pediatr. 2020 Sep 10;8:575. doi: 10.3389/fped.2020.00575 (PMC7511721; doi:10.3389/fped.2020.00575)
Supplement: Supplementary Video 1 — Proximal Coronary Ectasia on coronary angiography. [file Presentation_1.pptx]

## Slide 1
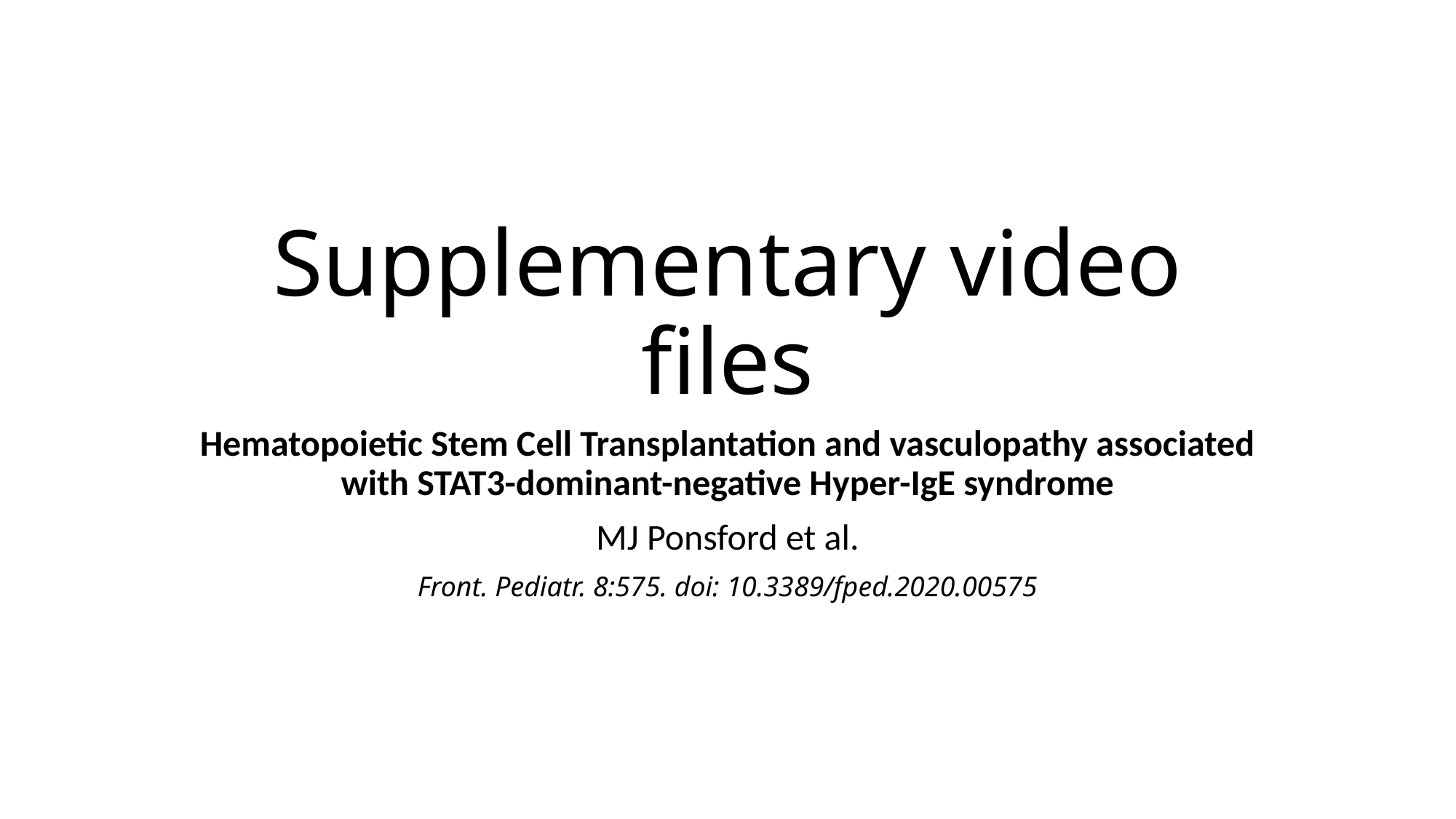

# Supplementary video files
Hematopoietic Stem Cell Transplantation and vasculopathy associated with STAT3-dominant-negative Hyper-IgE syndrome
MJ Ponsford et al.
Front. Pediatr. 8:575. doi: 10.3389/fped.2020.00575

## Slide 2
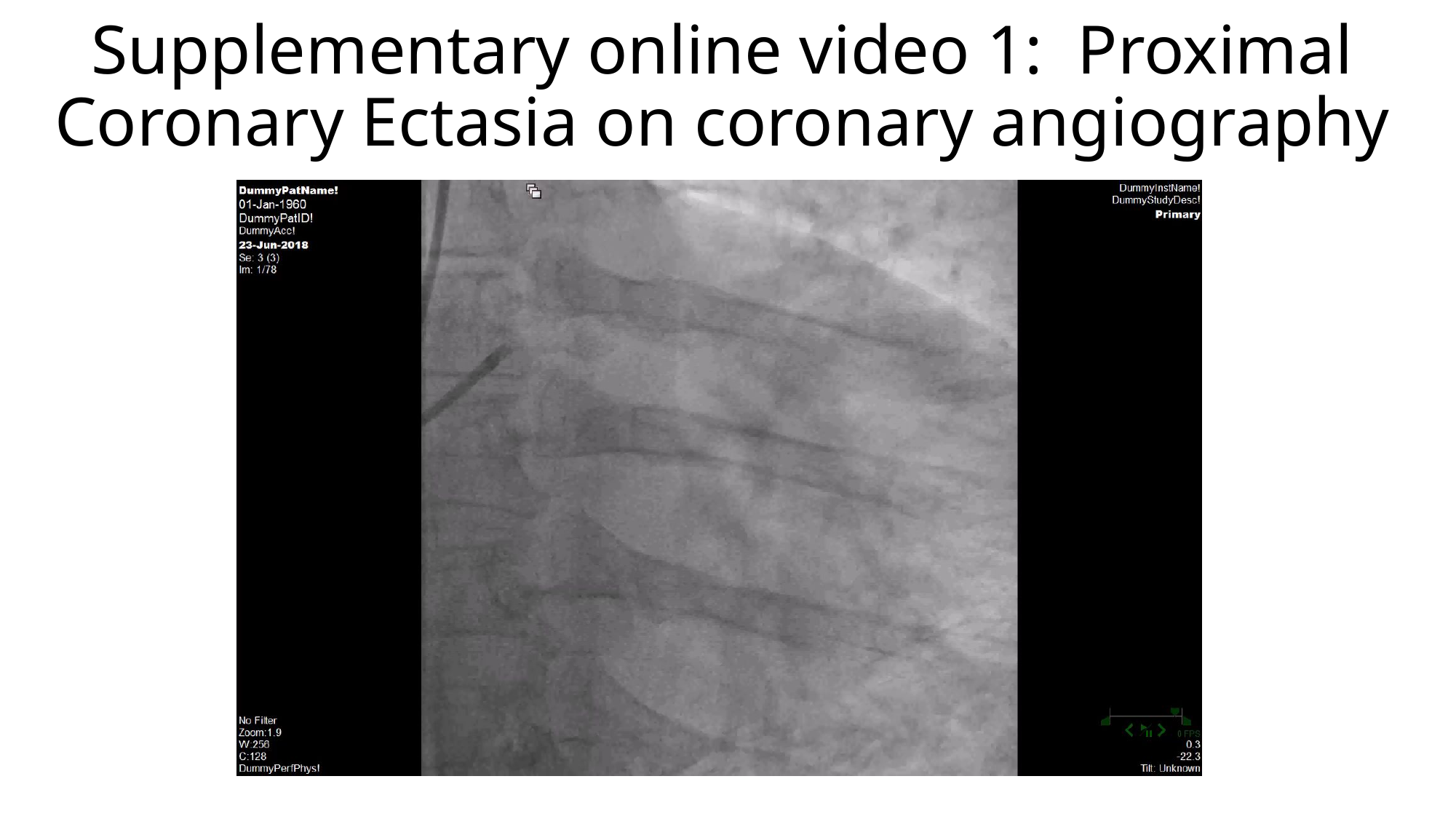

# Supplementary online video 1: Proximal Coronary Ectasia on coronary angiography

## Slide 3
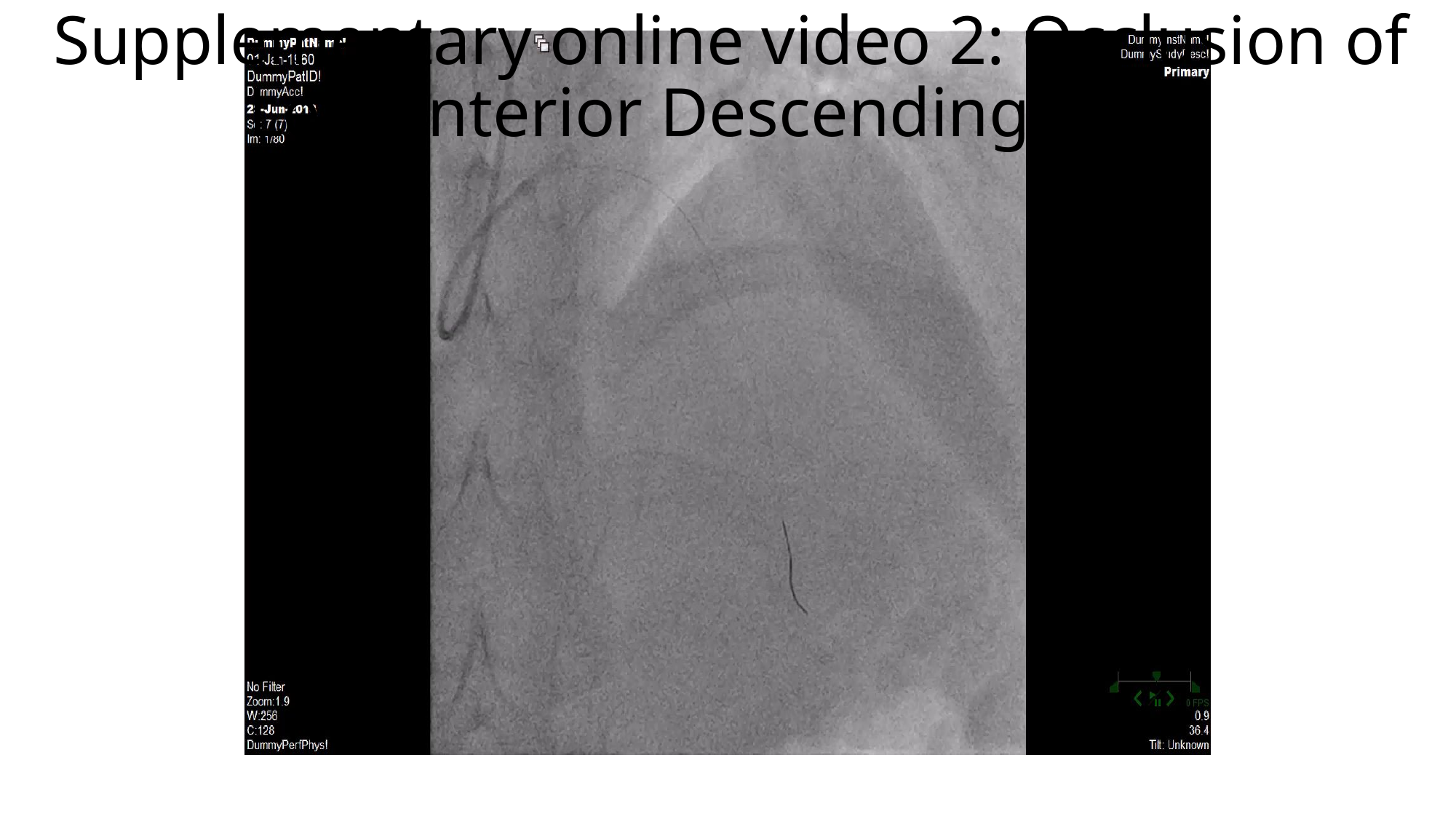

# Supplementary online video 2: Occlusion of Left Anterior Descending (LAD)

## Slide 4
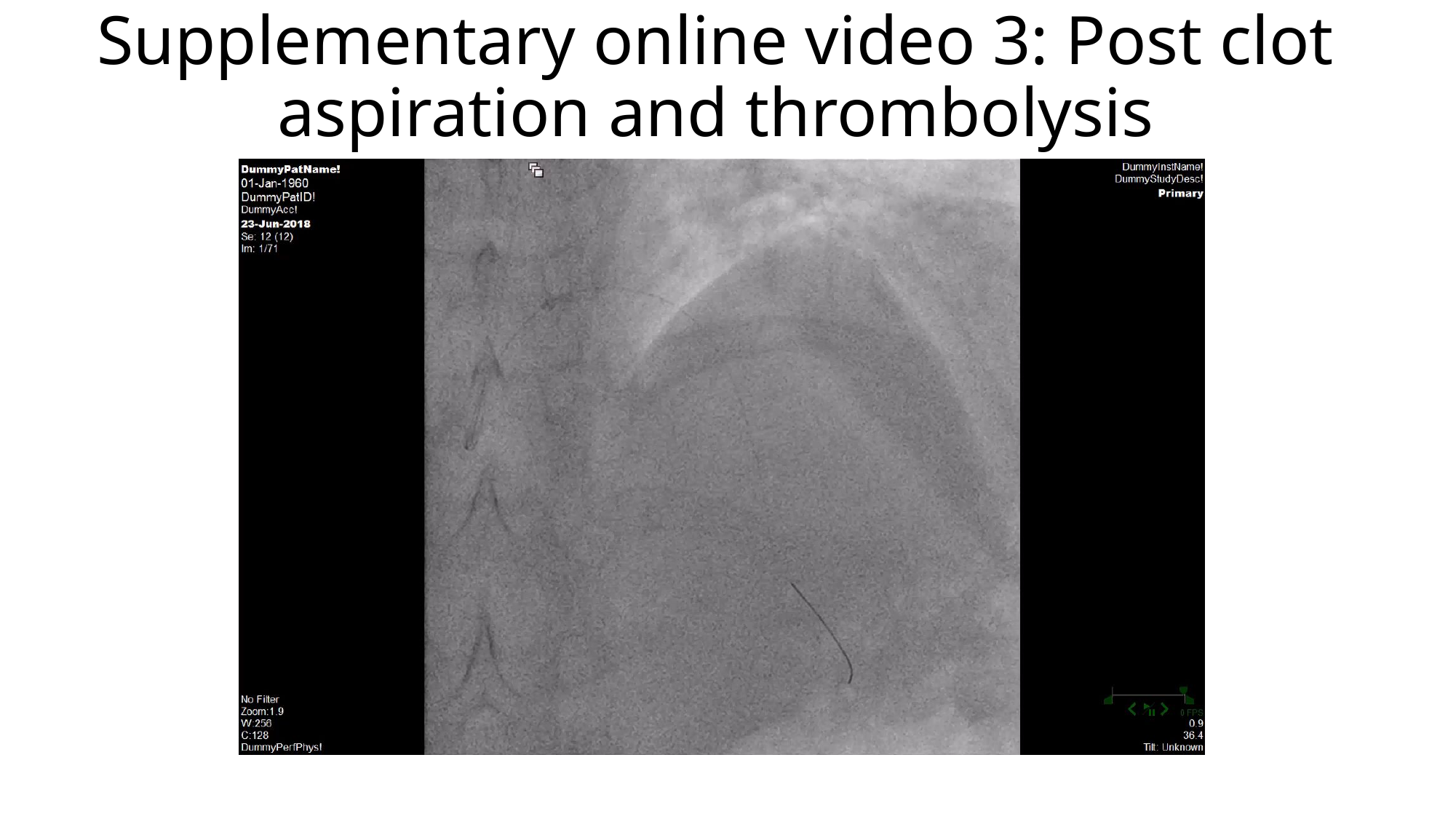

# Supplementary online video 3: Post clot aspiration and thrombolysis

## Slide 5
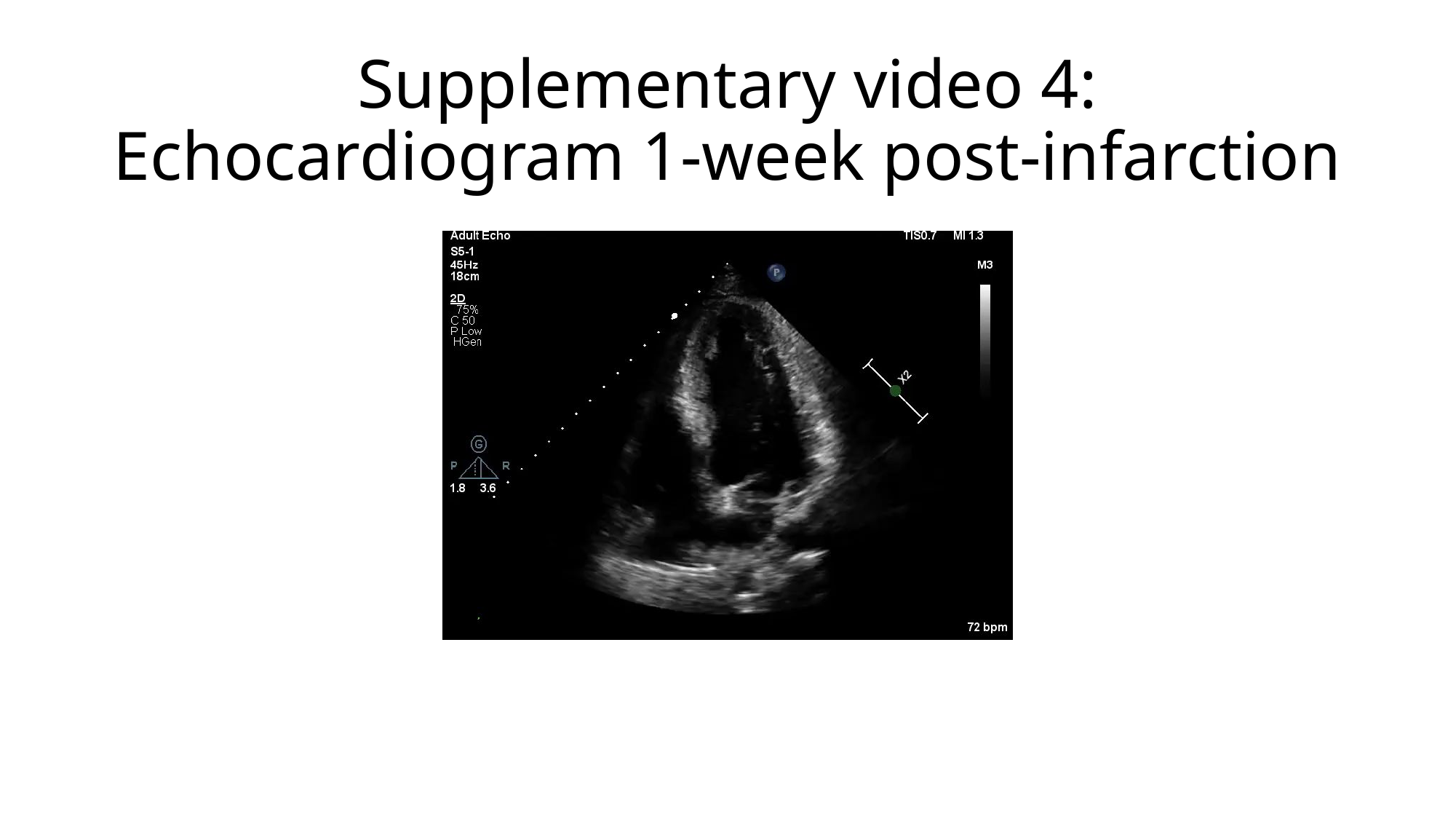

# Supplementary video 4: Echocardiogram 1-week post-infarction
